# Microbial community overlap between the phyllosphere and rhizosphere of three plants from Yongxing Island, South China Sea

**DOI:** 10.1002/mbo3.1048

**Published:** 2020-04-21

**Authors:** Lijun Bao, Wenyang Cai, Jianxi Cao, Xiaofen Zhang, Jinhong Liu, Hao Chen, Yuansong Wei, Xuliang Zhuang, Guoqiang Zhuang, Zhihui Bai

**Affiliations:** ^1^ Key Laboratory of Environmental Biotechnology Research Center for Eco‐Environmental Sciences Chinese Academy of Sciences Beijing China; ^2^ College of Resources and Environment University of Chinese Academy of Sciences Beijing China; ^3^ College of Biological Sciences and Biotechnology Beijing Forestry University Beijing China; ^4^ Tangshan Ecology and Environment Bureau Fengrun Branch Tangshan China; ^5^ Institute of Naval Engineering Design Naval Research Academy Beijing China

**Keywords:** bacteria, diazotrophs, fungi, microbiome, phyllosphere, rhizosphere

## Abstract

Phyllosphere and rhizosphere are unique and wide‐ranging habitats that harbor various microbial communities, which influence plant growth and health, and the productivity of the ecosystems. In this study, we characterized the shared microbiome of the phyllosphere and rhizosphere among three plants (*Ipomoea pes‐caprae*, *Wedelia chinensis*, and *Cocos nucifera*), to obtain an insight into the relationships between bacteria (including diazotrophic bacteria) and fungi, present on these host plants. Quantitative PCR showed that the abundances of the microbiome in the soil samples were significantly higher than those in the phyllosphere samples, though there was an extremely low abundance of fungi in bulk soil. High‐throughput sequencing showed that the alpha‐diversity of bacteria and fungi was higher in the rhizosphere than the phyllosphere samples associated with the same plant, while there was no obvious shift in the alpha‐diversity of diazotrophic communities between all the tested phyllosphere and soil samples. Results of the microbial composition showed that sample‐specific bacteria and fungi were found among the phyllosphere and rhizosphere of the different host plants. About 10%–27% of bacteria, including diazotrophs, and fungi overlapped between the phyllosphere and the rhizosphere of these host plants. No significant difference in microbial community structure was found among the tested rhizosphere samples, and soil properties had a higher influence on the soil microbial community structures than the host plant species.

## Graphical abstract

In this study, we characterized the shared microbiome of the phyllosphere and rhizosphere among three plants on Yongxing Island, South China Sea. We found that about 10%~27% of bacteria, including diazotrophs, and fungi overlapped between the phyllosphere and rhizosphere of these host plants. No significant difference in microbial community structure was found among the rhizosphere samples, and soil properties had a higher influence on soil community structure than on the host plant species.

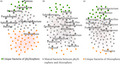

## 1 INTRODUCTION

The leaf surface, also known as the phyllosphere, is a large and extremely diverse habitat for terrestrial microorganisms. The estimated total leaf surface area in the word is approximately twice as large as the land surface area (Zimmerman & Vitousek, 2012). Many species of bacteria and fungi colonize leaf surfaces (where they mostly form aggregates) and the spaces inside the leaves (Vorholt, 2012). Phyllosphere bacteria are involved in large‐scale processes like the carbon and nitrogen cycles, including the fixation of nitrogen (N) and nitrification, thus affecting the health of individual plants (Fuernkranz et al., 2008;Lindow & Brandl, 2003). The interactions between plants and microbial communities are recognized as important drivers of the terrestrial ecosystems (van der Putten et al., 2013). The activities of the microbial community in the phyllosphere, as well as the rhizosphere, influence plant growth, and health and the productivity of the ecosystems. Indigenous *Methylobacterium* strains, for example, exhibit a growth‐promoting effect on agriculturally important crops, which is assumed to be based on plant hormone (such as cytokinins and auxins) production (Abanda‐Nkpwatt, Müsch, Tschiersch, Boettner, & Schwab, 2006;Innerebner, Knief, & Vorholt, 2011). Plants also influence the structure and function of microbial communities in the phyllosphere and rhizosphere. For example, differences in leaf morphology, chemistry, and physiology can affect the colonization of leaf surfaces by microorganisms, thus influencing the microbial communities (Vacher et al., 2016).

The rhizosphere is a battlefield where a complex community interacts with soilborne pathogens and influences the outcome of pathogen infections (Raaijmakers, Paulitz, Steinberg, Alabouvette, & Moenne‐Loccoz, 2009). A recent study in *Arabidopsis thaliana* revealed that *Sphingomonas* strains show a striking plant‐protecting effect by suppressing disease symptoms and diminishing pathogen growth (Innerebner et al., 2011). The mechanism by which rhizosphere microorganisms repress the incidence or severity of diseases caused by soilborne pathogens could include the production of antibiotic compounds, the consumption of pathogen stimulatory compounds, competition for nutrients and space, and the production of lytic enzymes (Doornbos, Loon, & Bakker, 2012). Microorganisms from the phyllosphere or rhizosphere, like the *Pseudomonas* and *Burkholderia*, which belong to the Proteobacteria phylum, or the fungi *Trichoderma* and *Gliocladium* from the Deuteromycetes class, can both affect plant growth and health (Innerebner et al., 2011;Raaijmakers et al., 2009). Berendsen, Pieterse, and Bakker (2012) found that a specific microbial community—consisting of both microflora and microfauna around the root (rhizosphere)—interacts with the host plant. Some plants, such as *chamomile*, *thyme*, and *eucalyptus*, provide not only nutrients for microorganisms but also contain unique antimicrobial metabolites in their exudates (Berg & Smalla, 2009).

The inoculum source of phyllosphere microbiota, including epiphytes and endophytes, is thought to vary due to the inherently open nature of the leaf environment. The source probably involves bacterial transmission by aerosols, insects, or soil (Maignien, Deforce, Chafee, Eren, & Simmons, 2014;Vorholt, 2012). Soil harbors an extraordinarily rich diversity of microbiota (Garbeva, Veen, & Elsas, 2004), and the soil surrounding these plants is the most likely origin for many of these organisms (Zarraonaindia et al., 2015). The analysis of microbial community composition in the rice revealed clear differences, both in terms of composition and complexity, between the rhizosphere and the phyllosphere, although some genera (like the *Methylobacterium*) were shared between the two (Knief et al., 2012). A recent study of the common grapevine (*Vitis vinifera*) microbiota showed that the root‐associated bacterial communities differed significantly from the aboveground communities, yet the microbiota of the leaves, flowers, and grapes shared a greater proportion of taxa with the soil communities than with each other. This suggests that soil may serve as a common bacterial reservoir for belowground and aboveground plant microbiota (Zarraonaindia et al., 2015). However, little is known about the overlapping microbial communities (both bacteria and fungi) between the phyllosphere and rhizosphere that play an important role in the ecology and evolution, and the promotion of plant growth and health.

Yongxing Island (Sansha City, Hainan Province, China) is a tropical island and the biggest coral island in the South China Sea. In our previous study, we described the distinct microbial communities of the phyllosphere associated with five plants on the island (Bao et al., 2019). The study showed that bacterial communities of the tropical forest soils are exceedingly distinct from those found in other ecosystem types (Delgado‐Baquerizo et al., 2018). From a functional viewpoint, there is a priori evidence that bacteria in tropical ecosystems may be more important than those in cooler areas, as the rates of nitrogen fixation estimated in a tropical forest, in all system components (soil and vegetation), are commonly thought to be among the highest of any natural ecosystem (Cleveland et al., 1999). There is also evidence to suggest that N_2_ fixation in the phyllosphere is the main mechanism for the addition of N in humid tropical ecosystems (Abril, Torres, & Bucher, 2005). The establishment of phyllospheric populations of diazotrophs has mostly been reported in several tropical plants (Fuernkranz et al., 2008;Goosem & Lamb, 1986). The abundance and composition of shared taxa between the phyllosphere and rhizosphere may differ from those in temperate ecosystems, but that is just an analogy. There is, however, mounting evidence that targeted manipulation of microorganisms can lead to more environmentally and economically sustainable production systems. To provide a certain theoretical basis for the above hypothesis and the construction of sustainable production systems, we collected the phyllosphere, rhizosphere, and bulk soil from three different host plant species (*Ipomoea pes‐caprae*, *Wedelia chinensis,* and *Cocos nucifera*) based on our previous study. We used high‐throughput amplicon sequencing to compare the microbial community composition, structure, and interaction, especially the shared microbes between the phyllosphere and rhizosphere, among the different host plant species in this tropical island.

## 2 MATERIALS AND METHODS

### 2.1 Site description and sample preparation

In December 2017, we chose three common and abundant plant species, *Ipomoea pes‐caprae* (IP), *Wedelia chinensis* (WC), and *Cocos nucifera* (CN), growing on the Yongxing Island, in the South China Sea (Hainan Ocean Administration, 1999). This island has a true tropical maritime monsoon climate, with an average annual precipitation of more than 1,300 mm. The rainy season begins in late May and ends in early November, and the dry season lasts from late November to early May of the following year. The mean annual temperature of the island was 26–27°C, with the highest temperatures occurring in May and June. The sampling area for the three collected plant species was within 500 m^2^, to avoid a possible effect of the environmental factors, like spatial distance. Twelve individual plants from each species were randomly chosen in this area. The sampled IP and WC were not covered by any plants, including the CN.

Rhizosphere soils collected by shaking off the soil attached to the roots, and mature stage leaves were collected for the phyllosphere samples. Bulk soil, or the surface soil that is not penetrated by roots, was also collected. The detailed sampling process of the phyllosphere samples was introduced in our previous study (Bao et al., 2019). Considering the heterogeneity of the tested leaves and soils, leaves and rhizosphere soil samples were collected from at least four plants, to form a composite sample from a 1.5 m × 1.5 m area. Three composite samples (more than 10 m apart) of the phyllosphere and rhizosphere were collected from each plant. Due to the wide space between CN trees (>5 m), the collecting area of CN was much bigger. At least four bulk soil samples were randomly selected from a 1.5 m × 1.5 m area and mixed to form one composite sample. Then, three composite samples were taken from each sampling site with more than 10 m distance. We collected all the phyllosphere, rhizosphere, and bulk soil samples as quickly as possible. And there was no rain in the week before the sampling. We assumed that micrometeorological conditions were similar across sites. To standardize conditions as much as possible, we only chose green, healthy‐looking, and intact leaves. Leaves from each plant were cut with a pair of sterilized scissors, and each leaf or soil sample was put in a Labplas bag, placed on ice, and quickly transported to the laboratory. Subsequently, each phyllosphere sample was immediately processed for DNA extraction, and each soil sample was divided into two parts: (a) One part was sieved through a 2.0 mm mesh and stored at 4°C for soil properties analysis, (b) and the second part was stored at −80°C for DNA extraction and molecular analysis.

### 2.2 DNA extraction and determination of soil properties

Thirty grams (more than five pieces) of leaves were placed in a 1,000‐ml sterile Erlenmeyer flask and filled with 500 ml sterile PBS buffer (pH 7.4, 1 × phosphate‐buffered saline buffer). Sonication, at 40 kHz frequency for 6 min was then performed in an ultrasonic cleaning bath to wash the microbial cells off the leaves, followed by shaking at 200 rpm for 20 min at 30°C. After shaking, sonication was continued for a further 3 min. To separate the microbial cells from the leaves, the cell suspensions were then filtered through a 0.22 µm × 50 mm sterile nylon membrane. Phyllospheric DNA was directly extracted from each of the collected membranes. Soil DNA was then extracted from 0.5 g of the fresh soil using the Fast®DNA SPIN Kit (MP Biomedicals) and was stored at −80°C.

Soil pH was measured using a pH meter (PB‐10, Sartorious) with a water‐to‐soil ratio of 2. Ammonium (NH_4_^+^‐N) and nitrate (NO_3_^−^‐N) were extracted from the soil samples with 2 mol/L KCl and determined using a Continuous Flow Analyzer (AA3, SEAL Analytical). Total carbon (TC) and total nitrogen (TN) were determined using the elemental analyzer (rapid cube, Elementar). Dissolved organic carbon (DOC) was determined using a Total Organic Carbon Analyzer (Vario TOC, Elementar). Soil moisture represented the quantity of water in the soil and was measured by a drying method. Soil properties were described in Table 1.

**TABLE 1:** Soil physicochemical properties in the rhizosphere and bulk soils

Samples	Moisture (%)	pH	NH_4_^+^‐*N* (mg/kg)	NO_3_^−^‐*N* (mg/kg)	DOC (mg/kg)	TC (mg/g)	TN (mg/g)
IP‐R	25.2 ± 3.61b	9.70 ± 0.01d	1.95 ± 0.01a	1.75 ± 0.01a	34.6 ± 7.17b	114 ± 0.31c	0.18 ± 0.03a
WC‐R	7.80 ± 0.05a	8.83 ± 0.01a	3.05 ± 0.04b	22.8 ± 0.17d	57.1 ± 0.01c	106 ± 0.92b	1.42 ± 0.11b
CN‐R	6.29 ± 0.84a	8.85 ± 0.01b	5.86 ± 0.07d	15.8 ± 0.06c	38.6 ± 0.19b	102 ± 1.44a	1.38 ± 0.31b
Bulk	26.0 ± 0.32b	9.20 ± 0.01c	4.60 ± 0.07c	2.41 ± 0.19b	21.3 ± 0.76a	115 ± 0.24c	0.15 ± 0.00a

Note

Data are means ± *SD* (*n* = 3). Different letters in each column indicate significant differences among the rhizosphere and bulk soil samples at *p* < .05.

Abbreviations: ammonium nitrogen; NO_3_^—^N, bulk soil; CN, Bulk, *Cocos nucifera*; DOC, dissolved organic carbon; IP, *Ipomoea pes‐caprae*; NH_4_^+^‐N, Nitrate nitrogen; pH, pH value; R, rhizosphere soil; TC, total carbon; TN, total nitrogen; WC, *Wedelia chinensis*.

### 2.3 Quantitative PCR analysis

The quantitative PCR (qPCR) thermal profiling of the fungal ITS region and bacterial 16S rRNA genes was performed using primers ITS1/ITS2 and 799F/1115R, respectively. Primer sets PolF/PolR were used to amplify a region of the *nifH* genes that is the DNA barcode marker for the molecular identification of diazotrophic bacteria (Poly, Monrozier, & Bally, 2001; Table A1). Quantitative PCR was performed using a CFX96 Optical Real‐Time Detection System (Bio‐Rad, Laboratories, Inc.) in a reaction volume of 20 µl containing 10.0 µl SYBR^®^Premix Ex Taq (Takara, Biotech), 0.5 µl of each primer (10 µM), and 1 µl of the DNA template. To obtain the standard curve, a *nifH* gene fragment was cloned into the pMD19‐T vector (Takara, Biotech) and subsequently transformed into *Escherichia coli* JM109 competent cells. The plasmid DNA was extracted by a Plasmid Purification Kit (Takara, Biotech). Plasmids containing the correct fragment length were selected, sequence‐verified, and then used as a template to generate a standard curve. Blanks were run with DNA‐free sterile water. Specific target gene amplification was confirmed by agarose gel electrophoresis and melting curve analysis. Each DNA sample was determined in triplicate. Amplification efficiencies ranged between 90.1% and 102.5% with an *R* ^2^ value of 0.991. The cycling conditions for the three genes were as follows: 40 cycles of 30 s at 95°C, annealing for 30 s at the temperatures in Table A1, and extension at 72°C for 30 s, and a final extension at 72°C for 8 min. A standard agarose gel electrophoresis was always performed at the end of a PCR run to verify the specificity of the amplification products.

### 2.4 High‐throughput sequencing and bioinformatics analysis

The 16S rRNA genes, ITS region, and *nifH* genes of the total DNA were sequenced using the Illumina paired‐end approach. PCR was performed in 50 µl reaction volumes containing 25 µl of Premix Taq DNA polymerase, 0.5 µl of the forward primer (20 mM), 0.5 µl of the reverse primer (20 mM), 23 µl of double‐distilled water (ddH_2_O), and 1 µl of the DNA template (20 ng total DNA). Illumina libraries were constructed using the MiSeq Reagent Kit v3, according to the manufacturer's instructions. High‐throughput, paired‐end sequencing was performed on the Illumina MiSeq PE250 (Illumina) platform.

Sequencing data analyses were performed using the free online platform of the Majorbio I‐Sanger Cloud Platform (www.i‐sanger.com). Raw sequence data with fastq files were quality‐filtered using Trimmomatic (Bolger, Lohse, & Usadel, 2014) and merged using FLASH (Magoc & Salzberg, 2011) with the following criteria. (a) Reads were truncated at any site receiving an average quality value below 20 over a 50‐bp sliding window. (b) Sequences whose overlap being longer than 10bp were merged according to their overlap with mismatch no more than 2bp. (c) Sequences for each sample were separated according to barcodes (exactly matching) and primers (allowing 2 nucleotide mismatching), and reads containing ambiguous bases were removed. Thereafter, operational taxonomic units (OTUs) were clustered with 97% similarity cutoff using UPARSE (version 7.1, https://drive5.com/usearch/) with a novel “greedy” algorithm that performs chimera filtering and OTU clustering simultaneously. To calculate community similarities, OTU‐based hierarchical cluster analysis with the unweighted pair group method of arithmetic means, a principal coordinates analysis (PCoA), based on the Bray–Curtis distance matrices, and an analysis of similarity (ANOSIM) were carried out, using the vegan package of R software (version 3.1.2). Significant differences between the absolute abundances and diversities of fungi, bacteria, and nitrogen‐fixing bacteria were determined by one‐way ANOVA using SPSS 16. The Mantel test was used to evaluate the correlation of environmental factors and microbial community structure (including fungal, bacterial, and diazotrophic community structures). Correlation analysis between environmental factors and α‐diversities was performed with the Spearman method, using the SPSS 16. Spearman's correlation was used to calculate significant differences in the dominant microbial taxon composition, alpha‐diversity, and phyllosphere variables. All six correlation networks were constructed using the Maslov–Sneppen procedure (Maslov & Sneppen, 2002;Wang et al., 2019) and visualized using Cytoscape 3.5.1. The statistical significance level for all the analyses was set at 0.05.

## 3 RESULTS

### 3.1 Abundances and diversities of bacteria, fungi, and diazotrophs between leaf and soil samples

**FIGURE 1:**
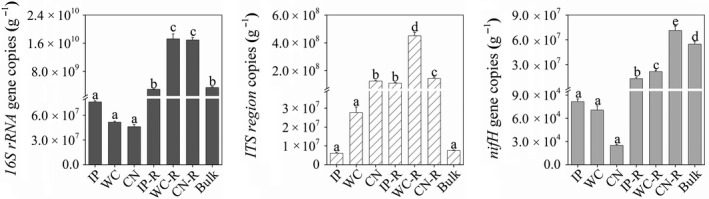
The copy numbers of the *16S rRNA* genes*, ITS* region, and *nifH* genes on the phyllosphere, rhizosphere, and bulk soils. Different lowercase letters above the columns indicate significant differences among all the samples at *p* < .05; IP indicates *Ipomoea pes‐caprae*, WC indicates *Wedelia chinensis*, CN indicates *Cocos nucifera*, R indicates rhizosphere soil, and Bulk indicates bulk soil

The abundances of bacteria, fungi, and diazotrophs among the phyllosphere, rhizosphere, and bulk soils of *Ipomoea pes‐caprae* (IP), *Wedelia chinensis* (WC), and *Cocos nucifera* (CN), detected by performing qPCR assays, were found to be different (Figure 1). The qPCR data showed that for three genes the copy numbers were mostly higher among the soil samples, than the phyllosphere samples (except for an extremely low abundance of copy numbers for the ITS region in the bulk soil). The abundance of diazotrophs, measured with the *nifH* genes, was lower than fungi and bacteria measured in the same habitat. Among the phyllosphere samples, CN harbored the highest ITS region copies, and the smallest 16S rRNA and *nifH* gene copies, especially when comparing it with the phyllosphere IP and WC, described in detail in our previous study (Bao et al., 2019). Among the rhizosphere samples, WC‐R and CN‐R had a higher abundance of diazotrophs, bacteria, and fungi compared to IP‐R (*p* < .05). Due to the very low abundances of fungi determined in all bulk soil replicates through qPCR assays and high‐throughput sequencing analysis, no results for fungi in the bulk soil samples are shown below.

To estimate sampling completeness, Good's coverage was determined (Rea et al., 2011). The high index of Good's coverage at a 97% similarity level of bacteria, fungi, and diazotrophs indicated that the sequencing depth contained almost all bacterial, fungal, and diazotrophic communities in all samples. The alpha‐diversities (Shannon index, Chao1, and Heip's evenness index) of bacteria, fungi, and diazotrophs were calculated with 22,264, 38,451, and 8,394 rarefied sequences per sample, respectively (Gotelli & Colwell, 2011;Heip, 1974;Table 2). Results showed that the rhizosphere and phyllosphere bacterial Shannon index, Chao1, and Heip's evenness index of IP were lower than for the other two plant species, with some of these indices having significant differences. Among the phyllosphere fungal communities, the Shannon index and Heip's evenness index of IP were significantly higher than that of WC and CN (Bao et al., 2019), while the fungal Shannon index and Heip's evenness index of the CN‐R rhizosphere were significantly higher than that of IP‐R and WC‐R. These three bacterial and fungal indices were mostly lower in the phyllosphere samples, compared to soil samples, although the Shannon index and Chao1 of fungi in rhizosphere IP‐R were slightly lower than that of phyllosphere IP. These results revealed that the sampled soils harbored a higher diversity, richness, and evenness of bacteria and fungi than the tested phyllosphere samples. Also, correlation analysis indicated that the rhizosphere Shannon index and Chao1 of the bacterial and fungal communities were negatively correlated with moisture and TC content, and positively correlated with soil NH_4_^+^‐N content (Spearman's correlation test, Table A2). The rhizosphere's Heip's evenness index of bacterial communities was positively correlated with the soil's NO_3_^−^‐N, DOC, and TN content, and negatively with the soil pH (Spearman's correlation test, Table A2).

**TABLE 2:** Alpha‐diversity of the bacterial community, fungal community, and diazotrophic community in the phyllosphere and soil samples

	Samples	Shannon	Chao1 (10^2^)	Heip's (10^–2^)	Coverage (10^–2^)
Bacteria	IP	2.13 ± 0.05a	4.98 ± 0.37a	2.29 ± 0.14a	99.7 ± 0.08a
	WC	4.40 ± 0.02c	12.8 ± 0.80ab	9.00 ± 1.01b	99.1 ± 0.25a
	CN	3.87 ± 0.08b	15.5 ± 2.23b	4.59 ± 0.48ab	98.7 ± 0.36a
	IP‐R	5.36 ± 0.05d	16.9 ± 0.09b	14.8 ± 0.98c	99.2 ± 0.06a
	WC‐R	6.28 ± 0.20f	35.2 ± 9.71c	18.8 ± 5.61c	98.1 ± 1.84a
	CN‐R	6.38 ± 0.06f	40.1 ± 3.09c	15.0 ± 2.01c	99.3 ± 0.17a
	Bulk	5.83 ± 0.23e	23.1 ± 6.68b	16.3 ± 2.89c	99.4 ± 0.50a
Fungi	IP	2.39 ± 0.07b	2.79 ± 0.16b	4.99 ± 0.15b	99.9 ± 0.01ab
	WC	1.35 ± 0.14a	2.90 ± 0.21b	1.32 ± 0.25a	99.9 ± 0.00ab
	CN	1.33 ± 0.02a	2.78 ± 0.41b	1.40 ± 0.08a	99.9 ± 0.02ab
	IP‐R	2.13 ± 0.38b	1.21 ± 0.08a	7.36 ± 3.66b	99.9 ± 0.00b
	WC‐R	3.49 ± 0.19c	5.44 ± 0.34c	6.80 ± 0.89b	99.8 ± 0.01a
	CN‐R	4.10 ± 0.04d	5.85 ± 0.04c	10.9 ± 0.01c	99.8 ± 0.09a
Diazotrophs	IP	1.85 ± 0.20a	1.27 ± 0.03a	4.84 ± 0.77a	99.8 ± 0.04b
	WC	4.30 ± 0.03c	2.21 ± 0.14b	35.6 ± 1.29d	99.8 ± 0.02b
	CN	4.71 ± 0.07d	4.62 ± 0.30c	26.3 ± 4.29bc	99.3 ± 0.05a
	IP‐R	1.96 ± 0.03a	0.98 ± 0.14a	7.91 ± 1.08a	99.8 ± 0.05b
	WC‐R	4.46 ± 0.19c	4.41 ± 0.07c	21.6 ± 3.80b	99.2 ± 0.11a
	CN‐R	2.69 ± 0.11b	2.65 ± 0.35b	7.50 ± 0.43a	99.3 ± 0.06a
	Bulk	4.30 ± 0.04c	2.69 ± 0.08b	27.9 ± 1.52c	99.8 ± 0.05b

Note

Different lowercase indicated a significant difference between samples.

Abbreviations: Bulk, bulk soil; CN, *Cocos nucifera*; IP, *Ipomoea pes‐caprae*; R, rhizosphere soil; WC indicates *Wedelia chinensis*.

The phyllosphere and rhizosphere diazotrophic Shannon index and Chao1 of IP were significantly lower than that of the other two plant species. However, there was no obvious shift in these three indices of diazotrophic communities between all the tested phyllosphere and soil samples (Table 2). Correlation analysis demonstrated that the Shannon index and Chao1 of the rhizosphere diazotrophic communities significantly were negatively correlated with soil pH and positively correlated with soil NO_3_^−^‐N, DOC, and TN content (Spearman's correlation test, Table A2).

### 3.2 Differences in the community structure between phyllosphere, rhizosphere, and bulk soil samples

**FIGURE 2:**
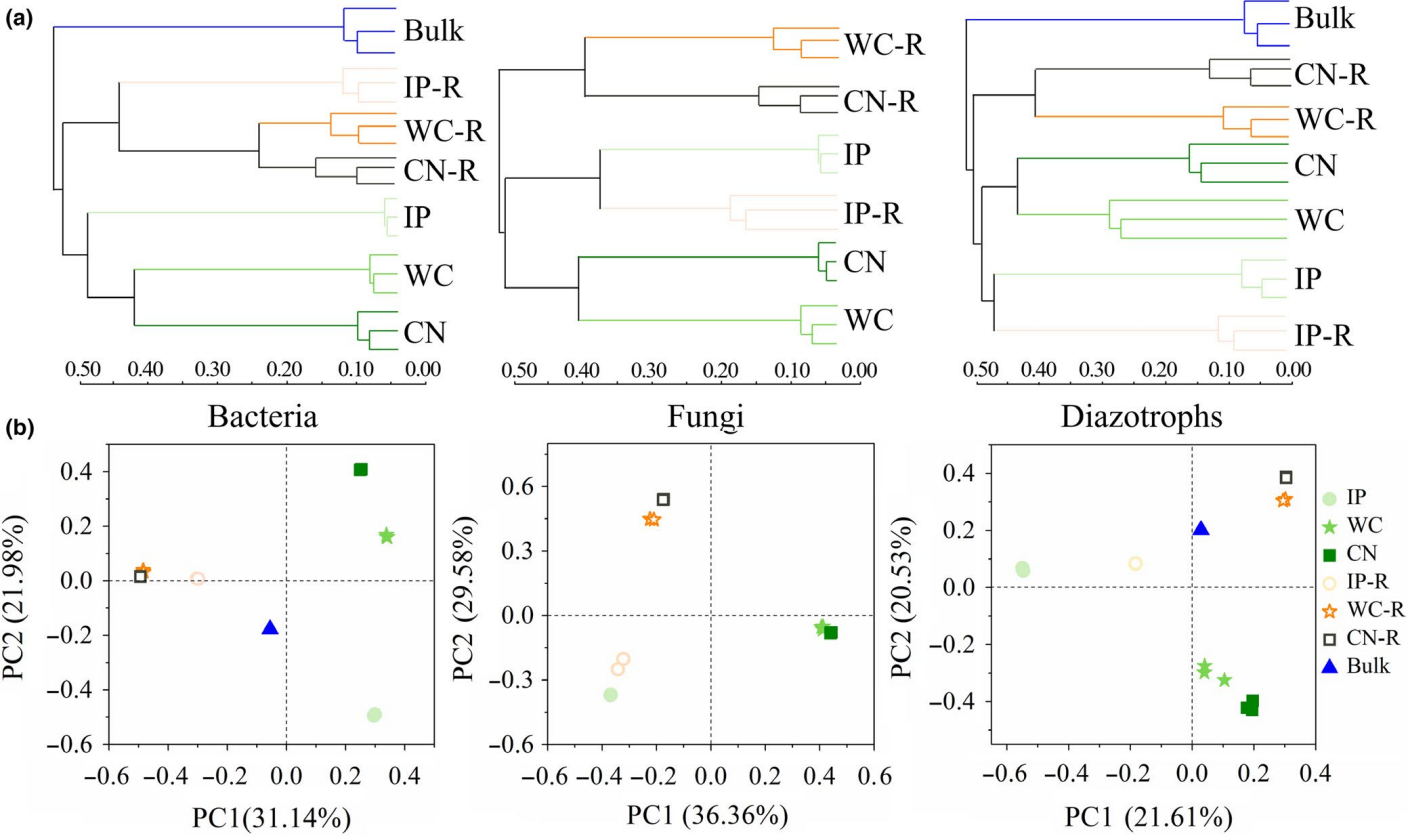
Hierarchical cluster analysis (a) and principal coordinates analysis (PCoA) (b) of bacterial, fungal, and nitrogen‐fixing bacterial community compositions in the sampled phyllosphere, rhizosphere, and bulk soils. IP indicates *Ipomoea pes‐caprae*, WC indicates *Wedelia chinensis*, CN indicates *Cocos nucifera*, R indicates rhizosphere soil, and Bulk indicates bulk soil

To compare the similarities and differences among the test samples, between the bacterial, fungal, and diazotrophic community structures, including phyllosphere (Bao et al., 2019), rhizosphere, and bulk soil samples, we used hierarchical clustering analysis, principal coordinates analysis (PCoA), and ANOSIM, based on the OTU composition. These analyses showed that fungal, bacterial, and diazotrophic communities were different between all the phyllosphere and soil samples (Figure 2). The dissimilarities in community structure between the different groups were calculated using ANOSIM. When comparing all the phyllosphere and rhizosphere samples of three plant species together, the results showed that there were significant differences in the bacterial, fungal, and diazotrophic communities among all the phyllosphere samples, rhizosphere versus bulk soil samples, and phyllosphere versus rhizosphere samples (*p* < .05, Tables A3, A4, A5). When comparing the phyllosphere and rhizosphere samples of only one of these plant species, we found no significant shifts in bacterial, fungal, and diazotrophic community structures were detected between the phyllosphere and rhizosphere samples from the same host plant (*p* > .05, Tables A3, A4, A5).

Therefore, the overlapping microbial community composition between the phyllosphere and rhizosphere of the same host plant became the focus of the next analysis in this study.

### 3.3 Ubiquity and bacterial dominance between the phyllosphere and the rhizosphere

**FIGURE A1:**
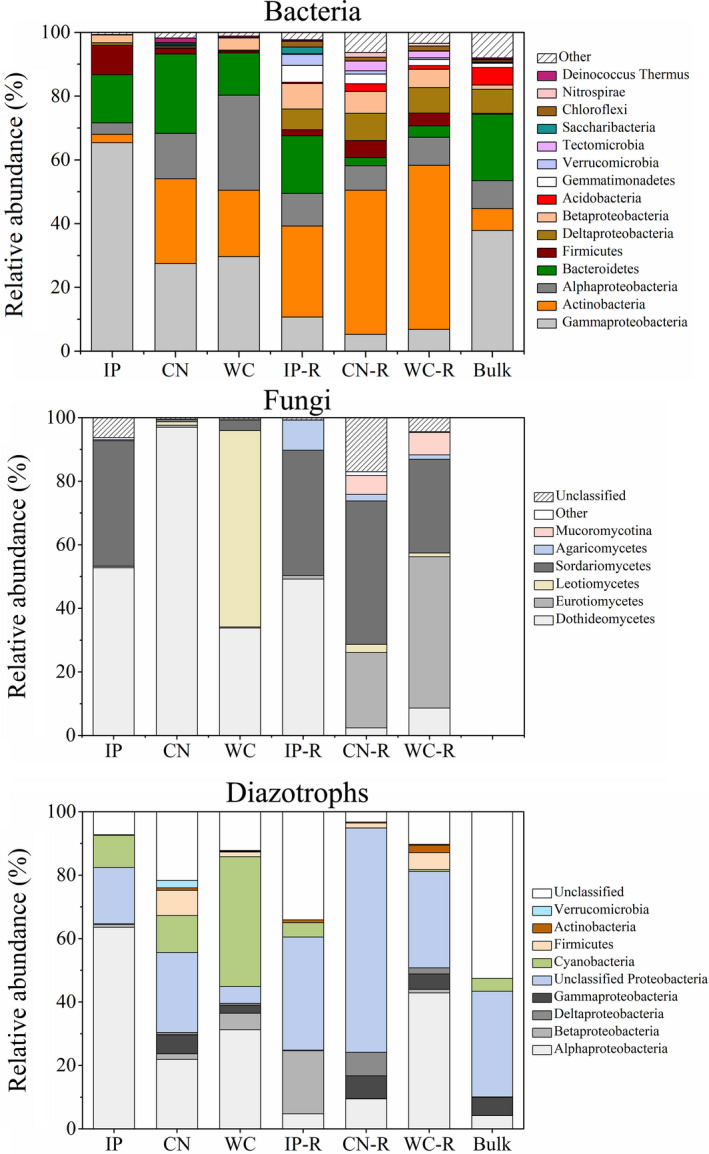
The relative abundance of bacteria, fungi, and diazotrophs at phylum/class level between phyllosphere, rhizosphere, and bulk soil samples. IP, *Ipomoea pes‐caprae*; WC, *Wedelia chinensis*; CN, *Cocos nucifera*; R, rhizosphere soil; Bulk, bulk soil

A total of 39 bacterial phyla and 961 bacterial genera were detected in the sampled phyllosphere, rhizosphere, and bulk soil samples. Gammaproteobacteria, Actinobacteria, Alphaproteobacteria, Bacteroidetes, Firmicutes, Deltaproteobacteria, and Betaproteobacteria were the dominant taxa in the phyllosphere, rhizosphere, and bulk soil samples, the sum of which was more than 90% of the total bacteria. The relative abundances of the major bacterial phyla in the test samples varied greatly between the phyllosphere, rhizosphere, and bulk soil samples (Figure A1). For example, Gammaproteobacteria dominated the phyllosphere samples (Bao et al., 2019), while the most abundant phylum in the rhizosphere samples was the Actinobacteria.

**FIGURE 3:**
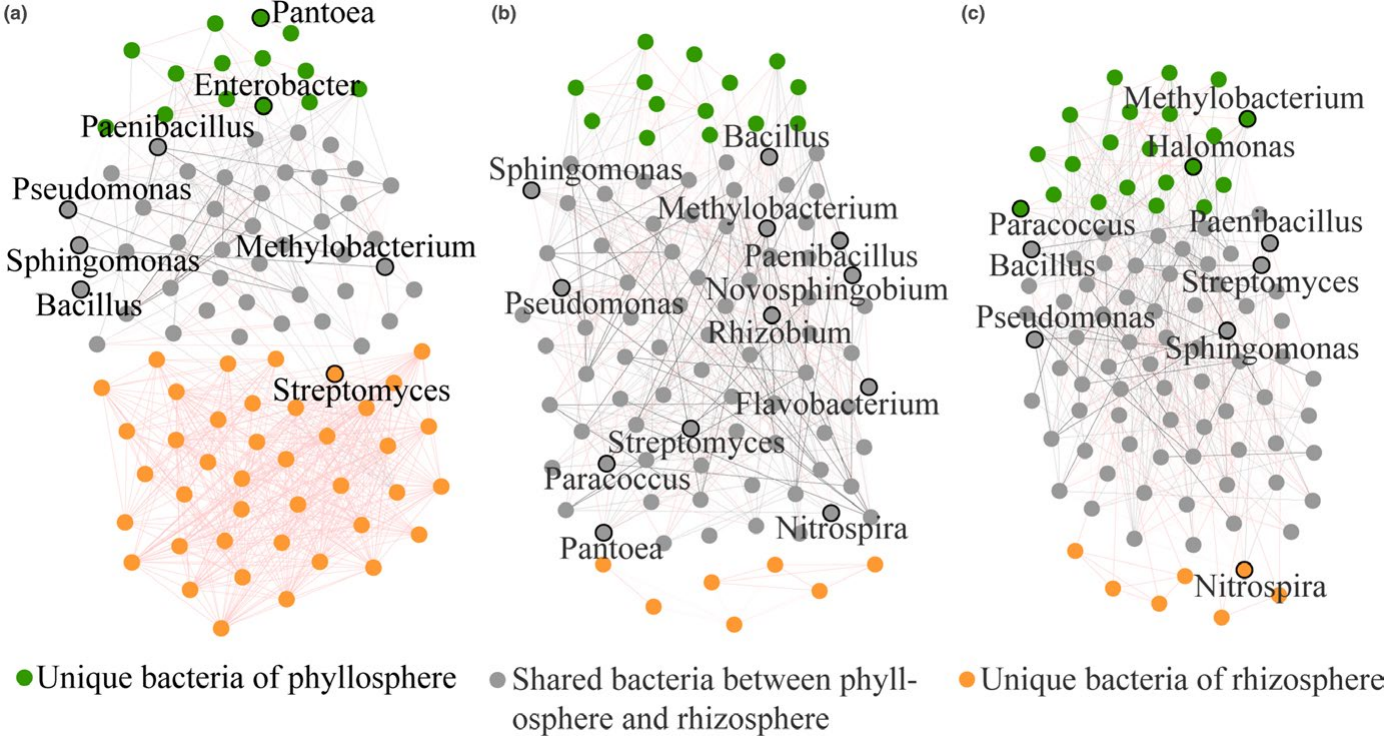
Bacterial 16S rRNA genera‐based correlation network for IP (a), WC (b), and CN (c). A node represents a genus. A connection stands for a strong (Spearman's *q* > 0.97 or *q* < −0.97) and significant (*p* < .01) correlation. Edge widths were scaled according to their weights, and edge colors indicated a positive (red) or negative (gray) correlation for the nodes they connect

**FIGURE 4:**
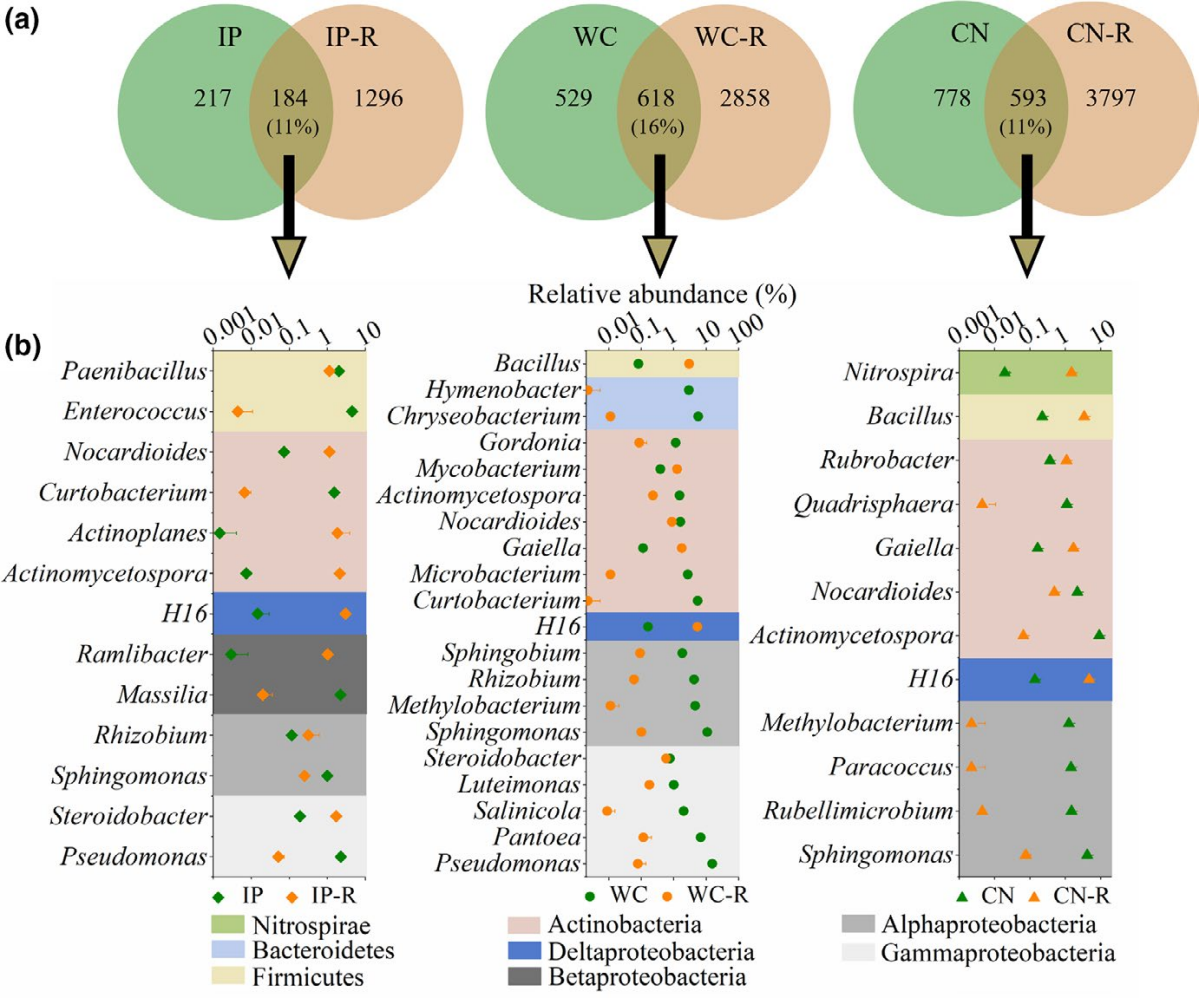
Venn diagrams showing the distribution of bacterial OTUs between the phyllosphere and rhizosphere samples of three different plant species (a). Relative abundances of shared genera between the phyllosphere and rhizosphere samples of three different plant species (b). IP indicates *Ipomoea pes‐caprae*, WC indicates *Wedelia chinensis*, CN indicates *Cocos nucifera*, and R indicates rhizosphere soil

On the genus level, network analysis was used to examine the relationship of all the bacteria between the phyllosphere and rhizosphere and then selected to show the significant (*p* < .01) correlations among the top 100 dominant genera (based on relative abundances) of the phyllosphere and rhizosphere, among the three plant species (Figure 3). And according to the relative abundances of the top 100 bacteria between the phyllosphere and rhizosphere samples, these significantly (*p* < .01) related bacteria were clustered into three distinct parts: unique bacteria of the phyllosphere, shared bacteria between the phyllosphere and rhizosphere, and unique bacteria of the rhizosphere (Figure 3). Shared bacteria had varied abundances throughout the leaf and rhizosphere soil samples, and some of the taxa had a high average relative abundance and a high prevalence in the leaf. These were likely able to adapt to the phyllosphere and were critical to the leaf microbiome. For example, the potential nitrogen‐fixing bacteria *Sphingomonas* dominated the phyllosphere (Figure 4b) with negative and positive interactions with the phyllospheric microbiome, among the three types of plants. Interestingly, many of the functional microorganisms were found to overlap between the phyllosphere and rhizosphere, including potential plant‐growth‐promoting bacteria, *Pseudomonas* and *Bacillus*; potential nitrogen‐fixing bacteria *Paenibacillus*, *Pantoea*, *Sphingomonas*, *Enterobacter*, and *Novosphingobium*; potential denitrifying bacteria *Halomonas*, *Streptomyces*, *Paracoccus*, and *Planococcus*; and potential ammonia‐oxidizing bacteria *Nitrosospira*. Furthermore, interactions between the unique bacteria of the phyllosphere or rhizosphere among the three plant types were found to be positive, indicating little competitive interaction between the unique microorganisms of the phyllosphere or rhizosphere, yet complex interactions existed in the shared bacteria between the phyllosphere and rhizosphere.

### 3.4 Ubiquity and fungal dominance between the phyllosphere and rhizosphere

Taxonomic classification revealed that 8 fungal phyla and 269 fungal genera were detected in the tested phyllosphere and rhizosphere samples. The presence of numerically dominant classes of Dothideomycetes, Eurotiomycetes, Leotiomycetes, Sordariomycetes, Agaricomycetes, and subphylum Mucoromycotina was detected in the phyllosphere and rhizosphere samples. The relative abundances of the aforementioned classes varied greatly between all the tested phyllosphere and rhizosphere samples. For example, Dothideomycetes were the most dominant class of phyllosphere CN (Bao et al., 2019). However, in the rhizosphere sample CN‐R, the most abundant class shifted to Eurotiomycetes and Sordariomycetes (Figure A1).

**FIGURE 5:**
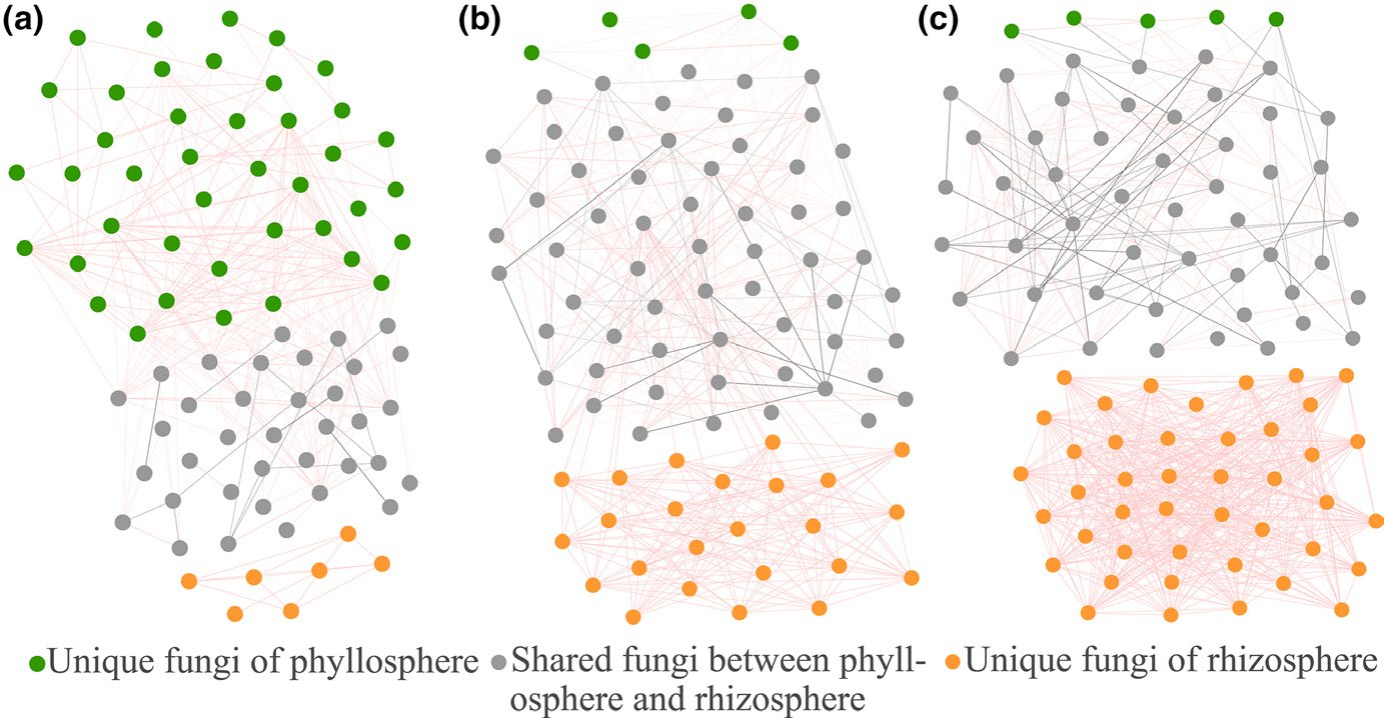
Fungal ITS region genera‐based correlation network for IP (a), WC (b), and CN (c). A node represents a genus. A connection stands for a strong (Spearman's *q* > 0.97 or *q* < −0.97) and significant (*p* < .01) correlation. Edge widths were scaled according to their weights, and edge colors indicated a positive (red) or negative (gray) correlation for the nodes they connect

At the genus level, network analysis was used to examine the relationship of all the fungi between the phyllosphere and rhizosphere and then selected to show the significant (*p* < .01) correlations among the top 100 dominant genera (based on relative abundances) between phyllosphere and rhizosphere among the three plant types (Figure 5). And according to the relative abundances of the top 100 fungi in the phyllosphere and rhizosphere samples, these significantly (*p* < .01) related fungi were clustered into three distinct, highly connected parts: unique phyllosphere fungi, shared fungi between the phyllosphere and rhizosphere, and unique rhizosphere fungi (Figure 5). Positive interactions were found between the unique fungi of phyllosphere or rhizosphere samples among the three plants and both negative and positive interactions in shared fungi. These results indicated that symbiotic interaction existed among the unique fungi and complex interaction existed in the shared fungi. Interestingly, among the three plant species, the proportion of unique fungi in the phyllosphere and rhizosphere was opposite to that of unique bacteria in the phyllosphere and rhizosphere. In IP, the number of unique fungi in the phyllosphere was much higher than in the rhizosphere, while the number of unique bacteria in the phyllosphere was lower than in the rhizosphere. In WC and CN, the number of unique fungi in the phyllosphere was lower than that in the rhizosphere; however, the number of unique bacteria in the phyllosphere was higher than that in the rhizosphere.

### 3.5 Shared bacterial and fungal communities between the phyllosphere and rhizosphere

Comparing the bacterial community composition of the phyllosphere (Bao et al., 2019) and rhizosphere samples, the proportions of shared OTUs between the phyllosphere and rhizosphere samples (phyllosphere and rhizosphere OTUs of the same plant species) were not different among the three plant types (Figure 4a). Among the top 40 bacterial genera (where the relative abundances of the taxa comprised more than 1.0% of the bacterial sequences in each sample), 13, 20, and 12 were shared between phyllosphere IP and rhizosphere IP‐R, phyllosphere WC and rhizosphere WC‐R, and phyllosphere CN and rhizosphere CN‐R, shown in Figure 4b, respectively. Among the shared bacteria at the genus level, *Sphingomonas*, *Actinomycetospora*, *Nocardioides,* and the H16 genus (from the Desulfurellaceae family) were present in the phyllosphere and rhizosphere samples of the three host plant species. The abundances of the four shared bacteria varied greatly among the phyllospheres and rhizospheres of the different host plant species. For example, the H16 genus was dominant in rhizosphere IP‐R, while its abundance was lower than 0.01% in phyllosphere IP. *Sphingomonas* was the most abundant genus in phyllosphere WC, while its abundance was lower than 0.1% in rhizosphere WC‐R, and *Actinomycetospora* was dominant in phyllosphere CN, while its abundance was lower than 0.1% in rhizosphere CN‐R. There were also unique dominant shared bacteria of the phyllosphere and rhizosphere at the genus level between the different host plant species. The comparison between phyllosphere IP and rhizosphere IP‐R revealed that phyllosphere IP was dominated by *Pseudomonas*, *Massilia*, *Enterococcus*, and *Paenibacillus*, while the rhizosphere IP‐R was dominated by *Steroidobacter* and *Actinoplanes*. When comparing the phyllosphere WC with the rhizosphere WC‐R, we could see that *Pseudomonas*, *Pantoea*, *Methylobacterium*, *Rhizobium*, *Curtobacterium,* and *Chryseobacterium* were the dominant genera in phyllosphere WC, while *Gaiella* and *Mycobacterium* were dominating the rhizosphere WC‐R. As for the comparison between phyllosphere CN and rhizosphere CN‐R, *Rubellimicrobium*, *Paracoccus*, *Methylobacterium,* and *Quadrisphaera* were the dominant genera in phyllosphere CN, while *Gaiella*, *Rubrobacter*, *Bacillus,* and *Nitrospira* were the dominant genera in rhizosphere CN‐R (Figure 4b).

**FIGURE 6:**
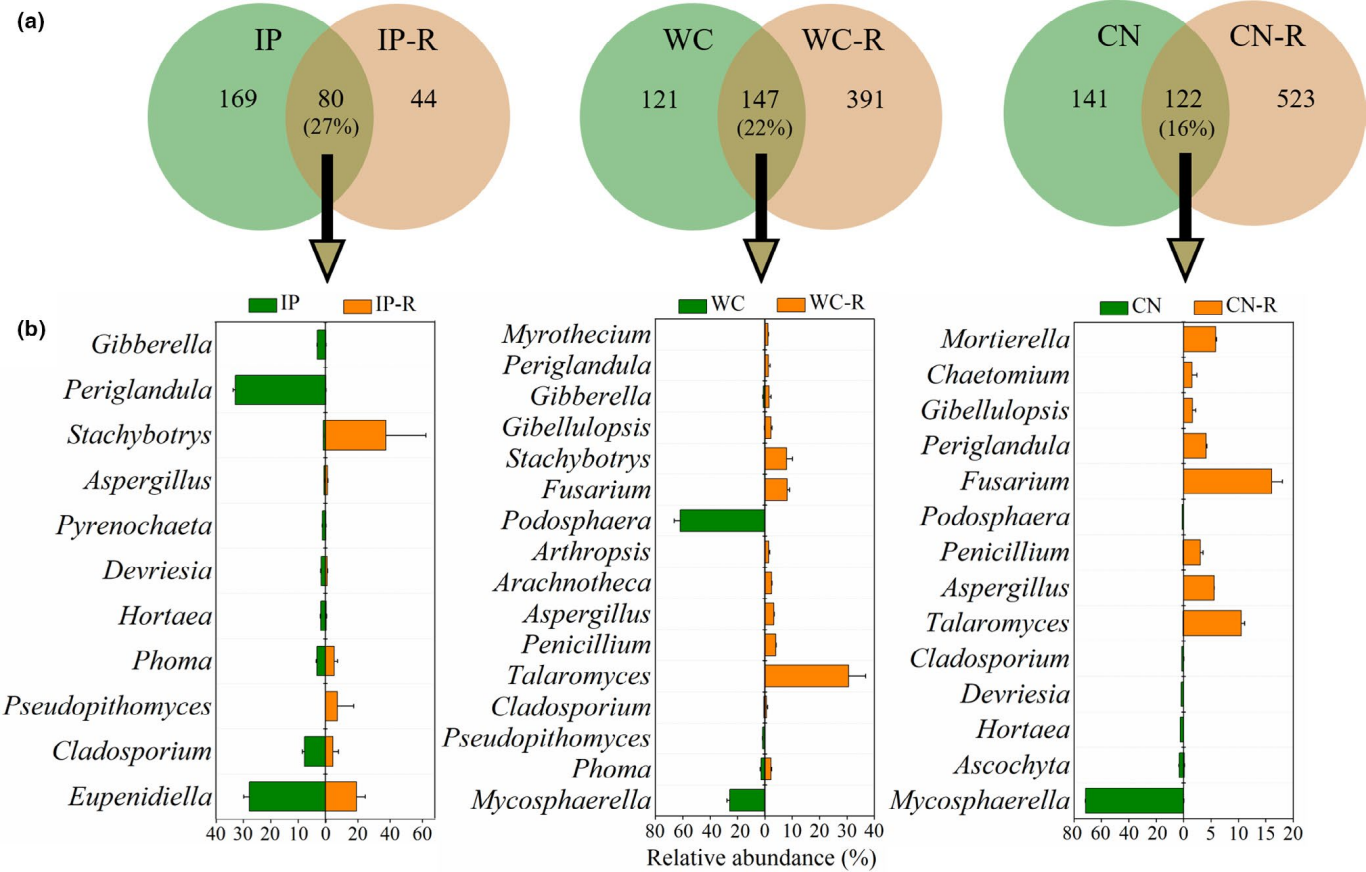
Venn diagrams showing the distribution of fungal OTUs between phyllosphere and rhizosphere samples of three different plant species (a). Relative abundances of shared fungal genera between the phyllosphere and rhizosphere samples of three different plant species (b). IP indicates *Ipomoea pes‐caprae*, WC indicates *Wedelia chinensis*, CN indicates *Cocos nucifera*, and R indicates rhizosphere soil

Comparing the fungal community composition of the phyllosphere (Bao et al., 2019) and rhizosphere samples, the proportions of shared OTUs between phyllosphere and rhizosphere (phyllosphere and rhizosphere OTUs of the same plant species) were not different among the three plant species (Figure 6a) but were higher than the shared bacterial OTUs. Among the top 33 fungal genera (relative abundances of the taxa comprising more than 1.0% of the fungal sequences in each sample), 11, 16, and 14 genera were shared between phyllosphere IP and rhizosphere IP‐R, phyllosphere WC and rhizosphere WC‐R, and phyllosphere CN and rhizosphere CN‐R, respectively. *Cladosporium*, *Aspergillus*, and *Periglandula* were all detected among the phyllosphere and rhizosphere samples from the three different host plant species. The relative abundances of the three shared fungi were found to be significantly different between the phyllosphere and rhizosphere samples, and even between the different host plants. For example, *Periglandula* was the most abundant genus in phyllosphere IP, while its abundance was lower than 0.1% in the rhizosphere IP‐R and phyllospheres WC and CN, but it shifted to a high abundance in rhizospheres WC‐R and CN‐R (Figure 6b). There were also unique dominant shared fungi among the phyllosphere and rhizosphere of the different host species. Between the phyllosphere IP and rhizosphere IP‐R, *Stachybotrys*, *Pseudopithomyces*, and *Phoma* occupied 49.63% of total reads in the rhizosphere, while their abundance was lower than 4% in the phyllosphere. A high abundance of *Eupenidiella* was detected both in the phyllosphere and rhizosphere of IP. When comparing phyllosphere WC with the rhizosphere WC‐R, the *Podosphaera* and *Mycosphaerella* genera occupied 87.35% of total reads in the phyllosphere, while their abundance was lower than 0.01% in the rhizosphere. *Talaromyces, Stachybotrys*, and *Fusarium* were the dominant genera with a relative abundance as high as 46.77% of all reads in the rhizosphere, instead of the 0.35% of all reads in the phyllosphere. Between the phyllosphere CN and rhizosphere CN‐R, *Fusarium* was the dominant genus in the rhizosphere, in comparison with a very low abundance in the phyllosphere. Instead, a relatively high abundance of *Mycosphaerella* was detected in the phyllosphere CN.

### 3.6 Diazotrophic community composition between the phyllosphere and rhizosphere samples

**FIGURE 7:**
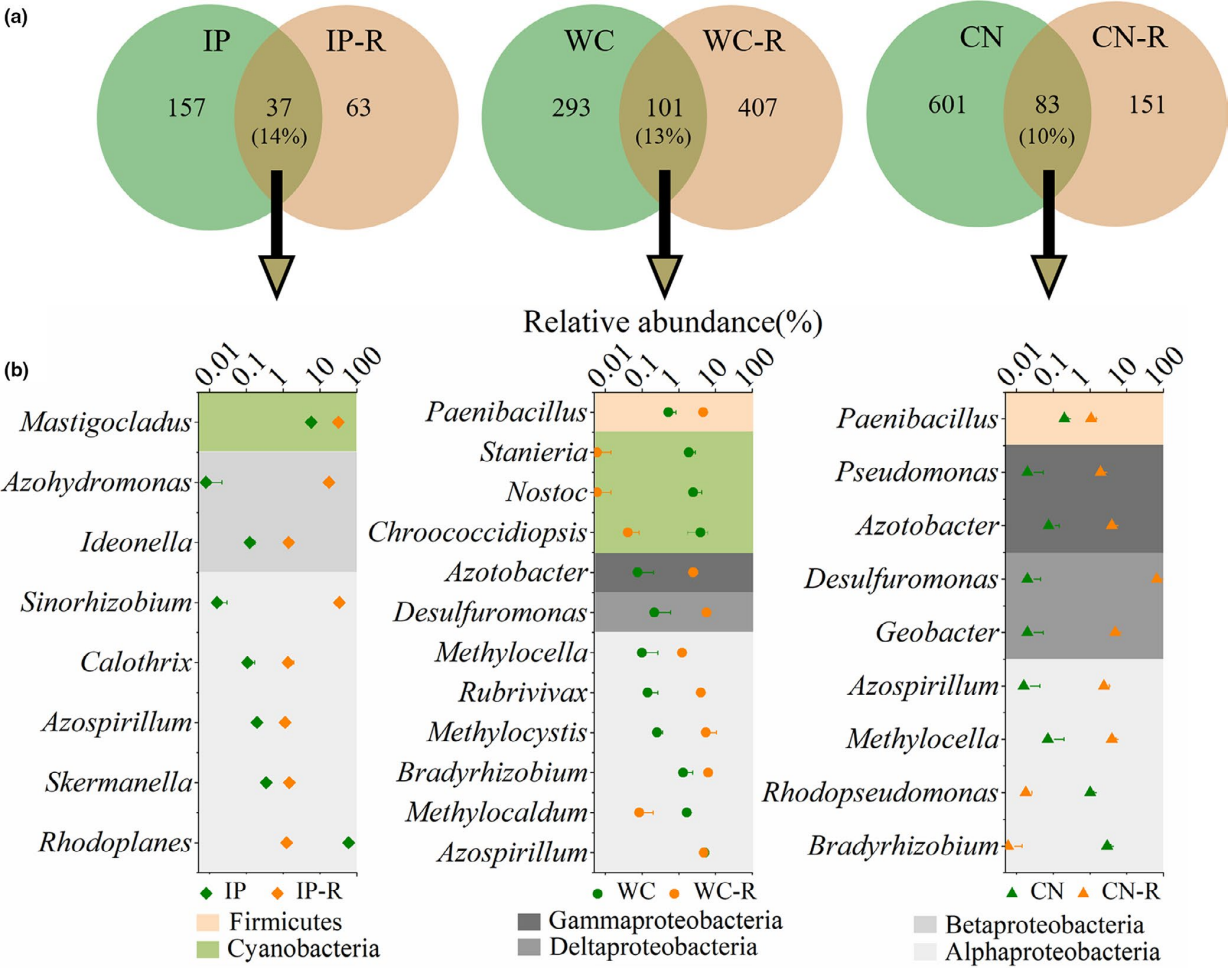
Venn diagrams showing the distribution of diazotrophic OTUs between phyllosphere and rhizosphere samples of three different plant species (a). Relative abundances of shared diazotrophs between the phyllosphere and rhizosphere samples of three different plant species (b). IP indicates *Ipomoea pes‐caprae*, WC indicates *Wedelia chinensis*, CN indicates *Cocos nucifera*, and R indicates rhizosphere soil

Comparing the diazotrophic community composition of the phyllosphere (Bao et al., 2019) and soil samples, a total of 9 phyla and 82 genera were detected in the sampled phyllosphere, rhizosphere, and bulk soil samples. Proteobacteria was the most abundant phylum in all the tested phyllosphere, rhizosphere, and bulk soil samples, compared to the Cyanobacteria phylum. Firmicutes, Actinobacteria, and Verrucomicrobia were also detected in the tested samples. The relative abundances of the different phyla varied greatly in the tested samples between the phyllosphere, rhizosphere, and bulk soil samples (Figure A1). At the OTU level, the proportions of shared diazotrophs between the phyllosphere and rhizosphere samples (phyllosphere and rhizosphere OTUs of the same plant species) were not markedly different among the three plant species (Figure 7a). With the top OTUs at the genus level (relative abundances of the taxa comprising more than 1.0% of the diazotrophic sequences in each sample), there were 8, 12, and 9 shared nitrogen‐fixing bacteria between the phyllosphere IP and rhizosphere IP‐R, phyllosphere WC and rhizosphere WC‐R, and phyllosphere CN and rhizosphere CN‐R, respectively (Figure 7b).

When comparing all the tested phyllosphere and rhizosphere samples of the three different plant species, we found a significant difference in the diazotrophic community structure (Table A5). Only *Azospirillum* was detected as a shared genus among the three groups of phyllosphere–rhizosphere samples, with a high relative abundance in both the WC and WC‐R samples. Most shared genera between the phyllosphere and rhizosphere samples belonged to the phylum Proteobacteria, while some shared genera to Cyanobacteria and Firmicutes. The composition of shared diazotrophs varied greatly between the phyllosphere and rhizosphere samples from the different host plant species. *Rhodoplanes*, assigned to Alphaproteobacteria, was the most abundant genus in phyllosphere IP, while its abundance was very low in rhizosphere IP‐R. *Mastigocladus*, *Sinorhizobium*, and *Azohydromonas*, the dominant genera in the rhizosphere IP‐R, occupied 82.85% of all reads. While comparing the phyllosphere WC with the rhizosphere WC‐R, we found that the relative abundance of the shared genera, *Chroococcidiopsis*, *Nostoc*, *Stanieria*, and *Methylocaldum*, occupied 9.91% of total reads in the phyllosphere, while the abundance was lower than 0.15% in the rhizosphere. *Bradyrhizobium*, *Paenibacillus*, *Methylocystis*, *Desulfuromonas*, and *Rubrivivax* were the dominant genera in the rhizosphere. Between the phyllosphere CN and rhizosphere CN‐R, *Bradyrhizobium* and *Rhodopseudomonas* were the most abundant genera in the phyllosphere, in comparison with a very low abundance in the rhizosphere. On the contrary, *Desulfuromonas* alone was detected in approximately 66.01% of all the reads in the rhizosphere, with a strictly low abundance in the phyllosphere. *Geobacter*, *Azotobacter*, and *Methylocella* occupied about 12.68% of the total reads in the rhizosphere CN‐R, while their abundance was lower than 0.20% in the phyllosphere CN.

### 3.7 Correlations between the environmental factors and the bacterial, fungal, and diazotrophic community structures

The Mantel test, based on the Bray–Curtis method, was used to examine the effect of environmental factors on the microbial community. Soil pH, moisture, NH_4_^+^‐N, DOC, TC, and TN content were positively correlated with the bacterial community in the rhizosphere samples. Among these correlations, the most significant was with the DOC content (Table 3, *R* = .9952, *p* < .05). The correlation between soil pH, NH_4_^+^‐N, DOC, and TN content and the rhizosphere fungal community structure was also found to be significant, and NH_4_^+^‐N had a higher correlation with the fungal community in the rhizosphere samples (Table 3, *R* = .90027, *p* < .05). Soil pH, moisture, NH_4_^+^‐N, NO_3_^−^‐N, DOC, TC, and TN content were positively associated with the diazotrophic community in the rhizosphere samples, among which the correlation with the soil TN content was the most significant (Table 3, *R* = .93948, *p* < .05). Among all the soil samples (including rhizosphere and bulk soil samples), bacterial communities were significantly correlated with soil NH_4_^+^‐N and DOC content, and diazotrophic communities were significantly associated with soil pH, moisture, NH_4_^+^‐N, DOC, TC, and TN content. NH_4_^+^‐N had a higher correlation with the bacterial (Table A6, *R* = .59223, *p* < .05) and diazotrophic (Table A6, *R* = .72654, *p* < .05) community, respectively.

**TABLE 3:** Mantel analysis of the relationship between environmental variables and microbial community structure of rhizosphere samples

Factors	Bacteria		Fungi		Diazotrophic bacteria	
	*r*	*p*	*r*	*p*	*r*	*p*
pH	.86322	**.041**	.75928	**.045**	.93472	**.036**
Moisture	.90696	**.021**	.58173	.078	.7989	**.026**
NH_4_^+^‐N	.99175	**.019**	.90027	**.007**	.84566	**.004**
NO_3_^−^‐N	.49719	.115	.5895	.105	.88322	**.033**
DOC	.9952	**.019**	.89493	**.02**	.87303	**.017**
TC	.96683	**.016**	.58278	.085	.62508	**.019**
TN	.88286	**.036**	.74962	**.035**	.93948	**.027**
All	.98967	**.016**	.90722	**.013**	.88855	**.011**

Note

Values in bold indicate significant differences at *p* < .05.

## 4 DISCUSSION

Previous studies have already demonstrated the composition of microbial communities among the phyllosphere and rhizosphere in different plants, but few studies investigated the shared microbiome between these two habitats. This question remains important, as the shared microbiome seems to be responsible for the connection between soil and plants and could play an important role in plant growth and health. Knief et al. (2012) observed the presence of the one‐carbon conversion processes in the rhizosphere, as well as in the phyllosphere. Chen et al. (2017) found 10 antibiotic resistance genes (ARGs) in the soil that also found their way onto the phyllosphere, giving reasons for possible concern. In this study, we systematically characterized the phyllosphere and rhizosphere microbiome of three different tropical plant species, growing on Yongxing Island in the South China Sea. We also revealed an interaction between plants and microorganisms, including bacteria, fungi, and nitrogen‐fixing bacteria.

Overall, the abundances and diversities of the microbiome in the soil samples (including rhizosphere and bulk soil samples) were higher than those in the phyllosphere, which was consistent with a previous study (Kim et al., 2012). The partial reasons behind this phenomenon may be explained by the following hypothesis. The phyllosphere is a relatively harsh habitat, characterized by rapid changes in water and nutrient availability, UV radiation intensity, and other environmental stresses (Beattie & Lindow, 1999). Roots are known to have a certain influence on the community structure (Dennis, Miller, & Hirsch, 2010;Shu, Pablo, Jun, & Danfeng, 2012), evidenced by a significant shift of microbial community structures between the rhizosphere and bulk soil samples, while no significant differences in the community structure were found in all rhizosphere samples of three different plant species. These results indicated that the soil properties had a greater influence than host plant species on soil community structure (including rhizosphere and bulk soil samples). Many studies found that soil dissolved carbon contents and N‐related soil properties were important factors in shaping the soil microbial community structure (Sun et al., 2016;Wang et al., 2017;Zeng et al., 2016). In this study, we also found that the microbial community structure in the rhizosphere and bulk soil samples was significantly affected by soil properties.

“Everything is everywhere, but the environment selects” is famously formulated in the Baas Becking hypothesis (de Wit & Bouvier, 2006), which is a good explanation for the different soil environments (different physical and chemical properties) affecting the distribution of microorganisms in our study. Our results also showed that the phyllosphere and rhizosphere samples of three plants had their unique microorganisms, which might be due to the different environmental factors, such as different oxygen concentration, temperature, between the leaf surfaces and rhizosphere soils, and strong UV radiation on the leaf surface (Beattie & Lindow, 1999). The unique and shared microbial composition of the phyllosphere and rhizosphere also varied greatly among different plant species, which might be related to the different leaf and rhizosphere microenvironments of different plant types. For example, different plant root exudates affected the microenvironment of rhizosphere microorganisms. Different plant species have different physiological structures and environments of leaves for phyllosphere microorganisms (Vacher et al., 2016). However, the shared microorganisms between the phyllosphere and rhizosphere were not necessarily the result of environmental selection and might be due to the overlap formed by the microorganisms in the vertical migration process. Microorganisms can migrate from roots to leaves, and Chi et al. (2005) found a dynamic infection process of *Rhizobia* beginning with surface colonization of the rhizoplane (especially at lateral root emergence), followed by endophytic colonization within roots, and then ascending endophytic migration into the stem base, leaf sheath, and leaves where they developed high populations. Moreover, precipitation events are considered to be one of the main abiotic factors that promoted the vertical migration of microorganisms throughout different habitats (Van Stan II et al., 2020). We assumed that micrometeorological conditions were similar across sites; however, we have no micrometeorological data to confirm this assumption. Therefore, there may be some unexplainable variability related to the unknown micrometeorological variability across sites. In our research, we also found that among the shared microorganisms between all the phyllosphere and rhizosphere samples from the different host species, several shared genera were ubiquitous. This might be because these shared microorganisms are generalists, which could transfer horizontally between distantly related plants and survive well (Frank, Saldierna Guzmán, & Shay, 2017). It was also possible that these shared microorganisms might be transmitted in plants through a vertical transfer of seeds and pollen, or horizontal transfer of soil, atmosphere, and insects (Frank et al., 2017).

In our study, the proportion of shared microorganisms between the phyllosphere and rhizosphere among three different plant species was a little bit smaller than what was generally observed in previous studies (Knief et al., 2012;Martins et al., 2013). One reason may be that one of the primer sets used in this study was selected to screen out chloroplast DNA, making the capture of any Cyanobacteria that may live on the leaves or in the soil hard. Nevertheless, a relative abundance of Cyanobacteria was detected with the diazotrophic primer sets in this study. Among the top 40 shared genera, only *Sphingomonas*, *Actinomycetospora*, *Nocardioides*, and the H16 genus (Desulfurellaceae family) were detected both in all the phyllosphere and rhizosphere samples from the different host species. The widely distributed *Sphingomonas* (water, soil, and plants) is a novel and abundant microbial resource for the biodegradation of aromatic compounds. It is also useful in environmental protection because it can degrade refractory pollutants such as PAHs and hexachlorobenzene isomers with a high catabolic capacity (Seo, Keum, & Li, 2009;White, Sutton, & Ringelberg, 1996). *Sphingomonas* species are often found in association with plants. Among the members of this genus, *Sphingomonas paucimobilis* has been shown to exhibit antagonism against the phytopathogenic fungus *Verticillium dahlia* (Berg & Ballin, 1994). Many strains have been isolated from the rhizosphere (Takeuchi et al., 1995). In our study, the abundance of the genus *Sphingomonas* was significantly higher in the phyllosphere than that in the rhizosphere, especially in association with the plant *Wedelia chinensis*. Due to the catabolic capacity and widespread distribution of *Sphingomonas*, the tropical plants in this study may play a significant role in environmental protection. The genus *Actinomycetospora* has been predominantly isolated from subtropical/tropical regions (Jiang et al., 2008). It is believed that the abundance or diversity of *Actinomycetospora* correlates with the climate (Yamamura et al., 2011a). Some strains belonging to the *Actinomycetes* genus have also been isolated from lichen samples (Yamamura et al., 2011b). *Nocardioides* is a common endophytic actinobacteria genus isolated from a diverse range of plant species, including those found in estuarine/mangrove ecosystems and algae and/or seaweeds of the marine ecosystems (Govindasamy, Franco, & Gupta, 2014). Interestingly, the *Pantoea* genus showed an extremely high abundance in the phyllosphere IP, but not in the associated rhizosphere samples, and was detected in the phyllosphere and rhizosphere samples of the WC and CN, with a very low abundance. *Pantoea* are frequently isolated from a wide range of ecological niches and have various biological roles as plant epi‐ or endophytes, biocontrol agents, plant‐growth promoters, or pathogens of both plant and animal hosts (De Maayer et al., 2012).

The fungal genera *Aspergillus*, *Periglandula*, and *Cladosporium* were shared between all the phyllosphere and rhizosphere samples among the different host plant species. The abundance of *Aspergillus*—a genus associated with severe asthma, allergic sinusitis, and bronchoalveolitis (O'Gorman, Fuller, & Dyer, 2009)—was higher in the rhizosphere, than in the phyllosphere, requiring great care. *Periglandula*, a fungal pathogen that can produce secondary metabolites, like loline and ergot alkaloids in *Periglandula*‐infected plants, especially Convolvulaceae (Panaccione, Beaulieu, & Cook, 2014), was abundant in the phyllosphere IP. Interestingly, the *Periglandula* genus was also found in the rhizosphere of WC and CN (in a certain quantity), indicating a higher number of possible host species in a diverse plant family. Thus, the ecological effects of *Periglandula* infection or the presence of ergot alkaloids in the associated host plants should not be neglected. Some *Cladosporium* strains, like the *Cladosporium sphaerospermum*, have been reported to be gibberellin (GA) producers (Hamayun et al., 2009). GA production is associated with plant growth and development. Only a few fungi associated with plants and/or soil have been reported as GA producers in previous studies (Kawaide, 2006;MacMillan, 2001). Interestingly, the *Cladosporium* genus showed a high abundance among the phyllosphere and rhizosphere samples of IP. This phenomenon might indicate that IP as a medicinal plant could inhibit the pathogenic infection of *Cladosporium*, which could promote growth nonetheless. This would be then similar to the mechanism by the *Pantoea* bacterial genus, also associated with IP, described earlier. Also, an extremely high abundance of *Mycosphaerella* was detected among the phyllosphere samples of CN, predicting a possible infection of CN by leaf spot (Crous & Wingfield, 1996), which would need to be prevented.

The diazotrophic community composition of the phyllosphere and rhizosphere associated with the three different host plants mainly consisted of Proteobacteria, followed by Cyanobacteria, which is similar to what has generally been observed in previous studies on tropical plants (Fuernkranz et al., 2008). The sequences affiliated with the *Azospirillum* genus were found in all the phyllosphere and rhizosphere samples among the different plant species. As free‐living diazotrophic bacteria, members of the *Azospirillum* genus are capable of promoting plant growth. Some studies reported her mechanisms for *Azospirillum* to promote plant growth, such as phytohormone and/or siderophore production, phosphate solubilization (Puente, Li, & Bashan, 2004), and several biologically active plant regulators, like nitric oxide and polyamines (Cassan & Diaz‐Zorita, 2016). Members of Cyanobacteria were absent or found at very low abundances in soil samples, except for the rhizosphere sample IP‐R, and bulk soil samples. It was interesting to see that a large amount of *Mastigocladus* was found only in the rhizosphere IP‐R. It might be insufficient to generalize that this genus is IP‐R‐specific because only a small subsection of the rhizosphere soil diversity on Yongxing Island was targeted in this study. However, its distinctive abundance was enough to conclude that there was a strong relationship between IP‐R and *Mastigocladus*. Chaparro, Badri, and Vivanco (2013) observed that compared to other microorganisms, Cyanobacteria significantly correlates with the highest number of root exudate compounds. Also, *Mastigocladus laminosus* is generally isolated from thermal environments, such as hot spring microbial mats (Estrella Alcamán, Fernandez, Delgado, Bergman, & Díez, 2015) and hot mineral soils (Soo, Wood, Grzymski, Mcdonald, & Cary, 2009). Therefore, the chemical and physical characteristics of IP‐R could be the reason behind the strong relationship between IP‐R and *Mastigocladus*, but this remains speculative.

## 5 CONCLUSIONS

In summary, microbiome abundances and diversities were higher in the soil than in the phyllosphere samples. Compared to *Wedelia chinensis* and *Cocos nucifera*, *Ipomoea pes‐caprae* had higher rhizosphere and phyllosphere bacterial alpha‐diversities. The fungal Shannon index and Heip's evenness index of the *Cocos nucifera* rhizosphere were significantly higher than those in the *Ipomoea pes‐caprae* rhizosphere and the *Wedelia chinensis* rhizosphere samples. Besides, there was no obvious shift in the three indices of the diazotrophic communities between all the tested soil samples. No significant differences were found in the community structure in the test rhizosphere soil samples. About 10%–27% of bacteria, diazotrophs, and fungi overlapped between the phyllosphere and rhizosphere of these different host plant species. The main reason behind the different phyllosphere community structure could be the plant species. However, soil properties had a higher influence on the soil microbial community structures than the host plant species.

## CONFLICTS OF INTEREST

None declared.

## AUTHOR CONTRIBUTION

**Lijun Bao**: data curation (lead); formal analysis (lead); methodology (lead); writing—original draft (lead); writing—review and editing (lead). **Wenyang Cai**: data curation (supporting); writing—original draft (supporting). **Jianxi Cao**: methodology (supporting). **Xiaofen Zhang**: funding acquisition (supporting); resources (supporting). **Jinhong Liu**: methodology (supporting). **Hao Chen**: funding acquisition (supporting); resources (supporting). **Yuansong Wei**: funding acquisition (supporting); software (supporting). **Xuliang Zhuang**: funding acquisition (supporting); supervision (supporting); writing—review and editing (supporting). **Guoqiang Zhuang**: funding acquisition (supporting); supervision (supporting); writing—review and editing (supporting). **Zhihui Bai**: funding acquisition (lead); investigation (lead); resources (lead); supervision (lead); writing—original draft (supporting); writing—review and editing (supporting).

## ETHICS STATEMENT

None required.

## Data Availability

All data are included in the main manuscript apart from the raw sequencing data files of bacteria, fungi, and diazotrophs which are available at the NCBI Sequence Read Archive under accession numbers SRP148402, SRP158738, and SRP144329, respectively.

## APPENDIX 1

**TABLE A1:** Primers used in this study

Primer name	Primer sequence (5’–3’)	Target gene	Annealing *T*°C
799F	AACMGGATTAGATACCCKG	bacteria *16S rRNA* genes	57
1115R	AGGGTTGCGCTCGTTG		
ITS1	CTTGGTCATTTAGAGGAAGTAA	ITS1 amplicon	55
ITS2	GCTGCGTTCTTCATCGATGC		
polF	TGCGAYCCSAARGCBGACTC	*nifH* genes	58
polR	ATSGCCATCATYTCRCCGGA		

**TABLE A2:** Correlation analysis between α‐diversity of rhizosphere soil microbial community and environmental factors based on Spearman's correlation test

	Factors	Shannon	Chao 1	Heip's
Bacteria	pH	−0.500	−0.500	**−1.000^**^**
	Moisture	**−1.000^**^**	**−1.000^**^**	−0.500
	NH_4_^+^‐*N*	**1.000^**^**	**1.000^**^**	0.500
	NO_3_^−^‐*N*	0.500	0.500	**1.000^**^**
	DOC	0.500	0.500	**1.000^**^**
	TC	**−1.000^**^**	**−1.000^**^**	−0.500
	TN	0.500	0.500	**1.000^**^**
Fungi	pH	−0.500	−0.500	0.500
	Moisture	**−1.000^**^**	**−1.000^**^**	−0.500
	NH_4_^+^‐*N*	**1.000^**^**	**1.000^**^**	0.500
	NO_3_^−^‐*N*	0.500	0.500	−0.500
	DOC	0.500	0.500	−0.500
	TC	**−1.000^**^**	**−1.000^**^**	−0.500
	TN	0.500	0.500	−0.500
Diazotrophs	pH	**−1.000^**^**	**−1.000^**^**	−0.500
	Moisture	−0.500	−0.500	0.500
	NH_4_^+^‐*N*	0.500	0.500	−0.500
	NO_3_^−^‐*N*	**1.000^**^**	**1.000^**^**	0.500
	DOC	**1.000^**^**	**1.000^**^**	0.500
	TC	−0.500	−0.500	0.500
	TN	**1.000^**^**	**1.000^**^**	0.500

Note:

**^**^**Significant differences at *p* < .05

**TABLE A3:** Analysis of similarities (ANOSIM) of bacterial communities between the samples

	*R*	*p*
Phyllosphere versus rhizosphere versus bulk	**.9908**	**.001**
Phyllosphere versus rhizosphere	**.9855**	**.002**
Rhizosphere versus rhizosphere	1	.063
Phyllosphere versus phyllosphere	**1**	**.007**
Rhizosphere versus bulk	**1**	**.04**
IP versus IP‐R	1	.114
WC versus WC‐R	1	.106
CN versus CN‐R	1	.117

Note

Values in bold indicate significant differences at *p* < .05.

**TABLE A4:** Analysis of similarities (ANOSIM) of fungal communities between the samples

	*R*	*p*
Phyllosphere versus rhizosphere	**.6289**	**.001**
Rhizosphere versus rhizosphere	1	.055
Phyllosphere versus phyllosphere	**1**	**.006**
IP versus IP‐R	1	.11
WC versus WC‐R	1	.097
CN versus CN‐R	1	.105

Note

Values in bold indicate significant differences at *p* < .05.

**TABLE A5:** Analysis of similarities (ANOSIM) of diazotrophic communities between the samples

	*R*	*p*
Phyllosphere versus rhizosphere versus bulk	**.7253**	**.001**
Phyllosphere versus rhizosphere	**.5643**	**.001**
Rhizosphere versus rhizosphere	1	.078
Phyllosphere versus phyllosphere	**1**	**.006**
Rhizosphere versus bulk	**1**	**.039**
IP versus IP‐R	1	.103
WC versus WC‐R	1	.084
CN versus CN‐R	1	.103

Note

Values in bold indicate significant differences at *p* < .05.

**TABLE A6:** Mantel analysis of the relationship between environmental variables and microbial community structure of soil samples

Factors	Bacteria		Diazotrophic bacteria	
	*r*	*p*	*r*	*p*
pH	.28015	.059	.42136	**.027**
Moisture	.22816	.126	.51669	**.021**
NH_4_^+^‐N	.59223	**.029**	.72654	**.003**
NO_3_^—^N	−.21944	.91	.23718	.095
DOC	.48731	**.02**	.61376	**.006**
TC	.321	.079	.4775	**.013**
TN	−.03226	.482	.45415	**.023**
All	.62237	**.009**	.70619	**.003**

Note

Values in bold indicate significant differences at *p* < .05.
